# Persistence of a Birth Cohort Effect in the US Among the Adult Homeless Population

**DOI:** 10.1001/jamanetworkopen.2024.52163

**Published:** 2024-12-26

**Authors:** Thomas Byrne, Kelly M. Doran, Randall Kuhn, Stephen Metraux, Maryanne Schretzman, Dan Treglia, Dennis P. Culhane

**Affiliations:** 1 School of Social Work, Boston University, Massachusetts; 2 Department of Emergency Medicine, New York University School of Medicine, New York; 3 Department of Population Health, New York University School of Medicine, New York; 4 Fielding School of Public Health, University of California at Los Angeles; 5 Biden School of Public Policy and Administration, University of Delaware, Newark; 6 Center for Innovation through Data Intelligence, City of New York, New York; 7 Center for State Health Policy, Rutgers University, New Brunswick, New Jersey; 8 School of Social Policy and Practice, University of Pennsylvania, Philadelphia

## Abstract

This cross-sectional study evaluates the persistence of a birth cohort effect among adults born between 1955 and 1965 in the US homeless population.

## Introduction

Older adult homelessness is increasing in the US. Research^1^ published more than a decade ago using 1990 to 2010 decennial Census data suggested this is due to a birth cohort effect. Persons born between 1955 and 1965 have composed a disproportionate share of the single adult homeless population since the 1980s.^1^ We have thus far lacked information about whether this trend has persisted since 2010. This is a critical gap, as advancing age combined with the complex health needs of older homeless adults^2,3^ will have serious implications for health care and social service systems. Here, we document the persistence of this cohort effect to the year 2020 and the attendant emergence of over-65 homelessness as a large-scale phenomenon.

## Methods

This cross-sectional study used special tabulations provided by the US Census Bureau to describe changes in the age distribution of the sheltered adult male population from 1990 to 2020. These tabulations include nationwide and state-level estimates of the number of adult men enumerated by the Census Bureau in emergency shelters in discrete age groups specified by the Census Bureau that are not all equally sized. This study used aggregate, nonidentifiable data, and was thus deemed exempt from institutional review board approval and the need for informed consent, in accordance with 45 CFR §46. We followed the Strengthening the Reporting of Observational Studies in Epidemiology (STROBE) reporting guideline. Descriptive analyses were conducted in R Version 4.3.1 (R Project for Statistical Analysis) in June 2024.

## Results

**Figure 1.**
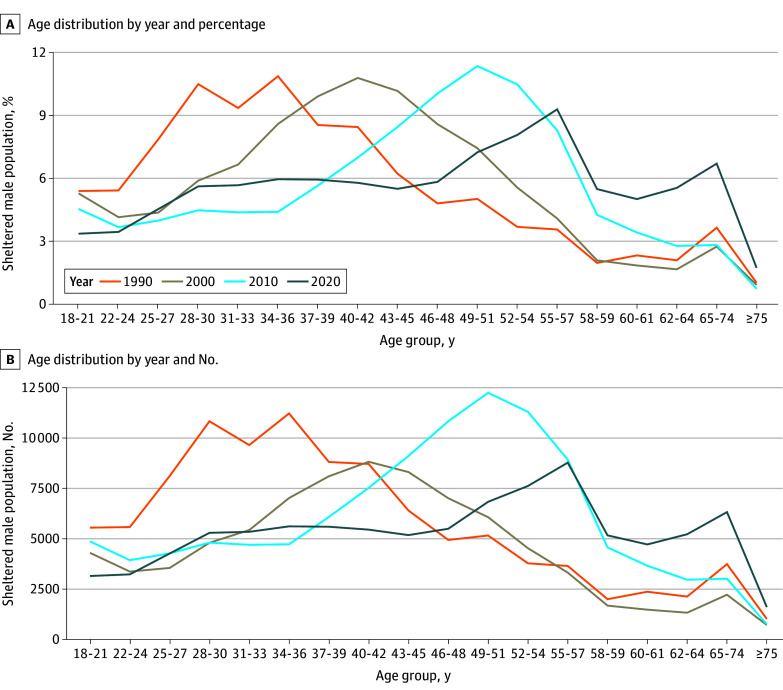
Age Distribution of Adult Male Shelter Users in the US, 1990 to 2020 Age groups were defined by the US Census Bureau and are not equally sized. Age groups below the top-coded 75 years or older age group can include 2 discrete years of age (58-59 or 60-61); 3 discrete years of age (22-24, 25-27, 28-30, 31-33, 34-36, 37-39, 40-42, 43-45, 46-48, 49-51, 52-54, 55-57, or 62-64); 4 discrete years of a age (18-21); or 9 discrete years of age (65-74). These differences account, for example, for the apparent peak in the figure for the age group 65 to 74 years (which in actuality is an artifact of the larger number of years in this age category). The primary cohort effect described in this paper is shown by the movement of the largest peaks in the sheltered male population rightward (toward higher ages) over time.

**Figure 2.**
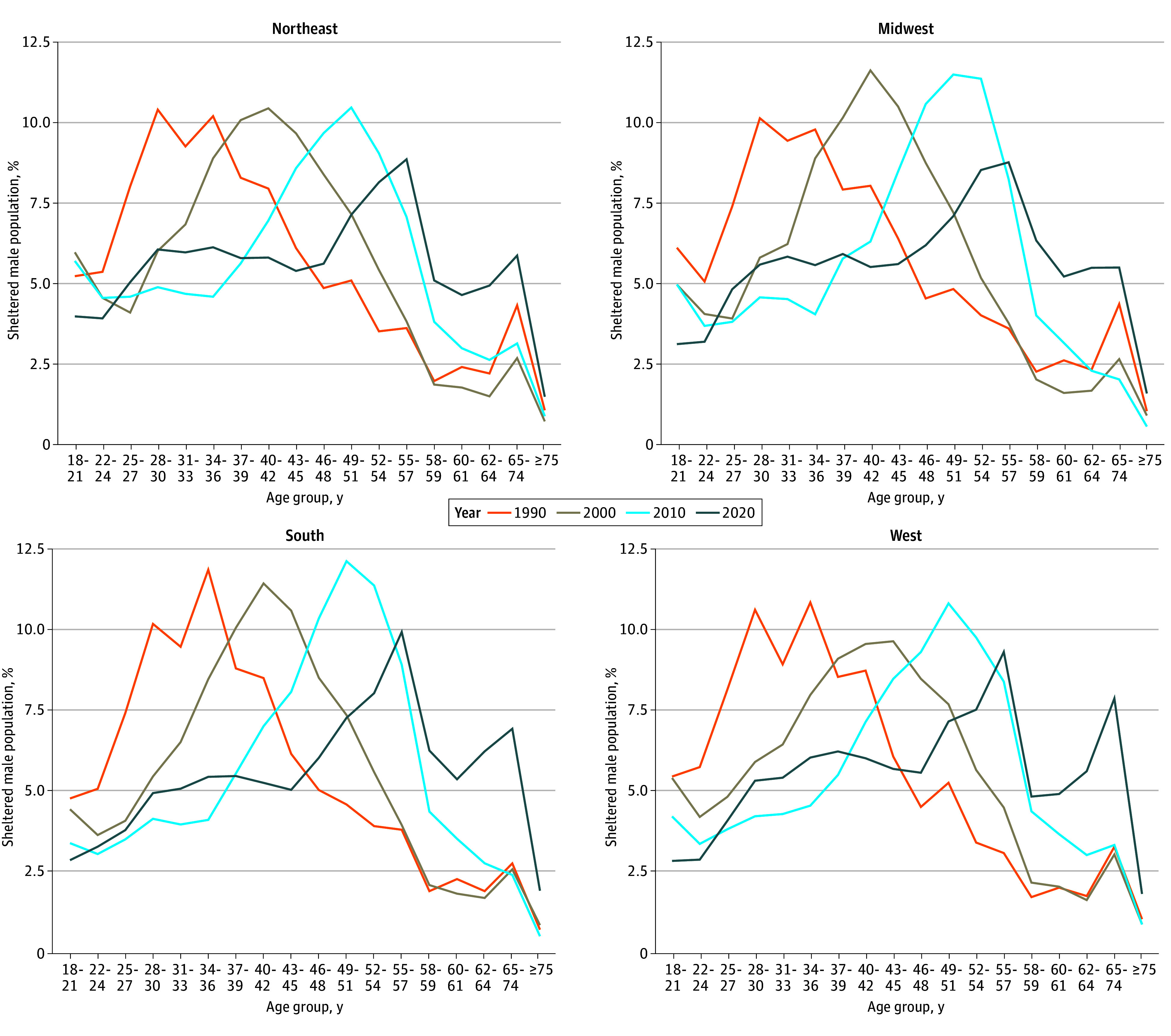
Age Distribution of Adult Male Shelter Users in the US by Census Bureau Defined Region, 1990 to 2020 Age groups were defined by the US Census Bureau and are not equally sized. Age groups below the top-coded 75 years or older age group can include 2 discrete years of age (58-59 or 60-61); 3 discrete years of age (22-24, 25-27, 28-30, 31-33, 34-36, 37-39, 40-42, 43-45, 46-48, 49-51, 52-54, 55-57, or 62-64); 4 discrete years of age (18-21); or 9 discrete years of age (65-74). The Census Bureau–defined regions include the following states: Northeast (Connecticut, Maine, Massachusetts, New Hampshire, New Jersey, New York, Pennsylvania, Rhode Island, and Vermont); Midwest (Illinois, Indiana, Iowa, Kansas, Michigan, Minnesota, Missouri, Nebraska, North Dakota, Ohio, South Dakota, and Wisconsin); South (Alabama, Arkansas, Delaware, District of Columbia, Florida, Georgia, Kentucky, Louisiana, Maryland, Mississippi, North Carolina, Oklahoma, South Carolina, Tennessee, Texas, Virginia, and West Virginia); and West (Alaska, Arizona, California, Colorado, Hawaii, Idaho, Montana, Nevada, New Mexico, Oregon, Utah, Washington, and Wyoming).

Figure 1 shows the age distribution of the nationwide sheltered adult male population from 1990 to 2020. Three findings are clear. First, across all decades, the peak age groups in the distribution (34-36 in 1990; 40-42 in 2000; 49-51 in 2010; and 55-57 in 2020) are composed of persons born from the mid-1950s to mid-1960s, demonstrating a persistent cohort effect. Second, older persons composed a substantially larger share of the sheltered population in 2020 relative to earlier years; in 2020, those aged 60 years and above accounted for 18.8% (17 950 of 95 251) of the sheltered adult male population compared with 7.1% in 2000 (5830 of 82 420), a roughly 3-fold increase in absolute numbers. Figure 2 presents the same data stratified by the 4 US Census–defined regions. The national aging trend is consistent across regions; in 2020, people aged 60 years and above accounted for 17.0% (6011 of 35 431), 17.8% (2166 of 12 153), 20.6% (4093 of 19 832), and 20.4% (5680 of 27 835) of the sheltered male adult population in the Northeast, South, Midwest, and West, respectively. Finally, in all years there is a sizeable drop-off in the number of sheltered men after the age of 60 years.

## Discussion

This study provides evidence that a previously known age-cohort effect in the homeless population—which is attributable to this cohort’s elevated relative risk of homelessness^1^—has persisted in the decade since it was first documented, with significant implications for health and health care. With the bulk of this cohort being aged in their 60s by the end of this study’s observation period, the share of the homeless population aged 60 years and older in 2020 was 2.6 times higher than in 1990. The similarity of the progression of this age-cohort effect across regions suggests that communities throughout the US are likely to be affected by the myriad health needs faced by an aging homeless population. Additionally, the observed drop-off in the homeless population after age 60 years is likely due to myriad factors including accelerated aging^2^ and premature mortality, entry into nursing homes,^4^ or receipt of Social Security or pensions that may facilitate access to housing.

The study is limited by its focus on the sheltered male population. As the available data do not differentiate by household type, we use this group as a proxy for the single adult homeless population; most homeless adult men are individuals and not part of a family with children.^5^ The observed age cohort effect does not appear to exist for homeless families; it is unclear whether our findings apply to single homeless women and single unsheltered adults.

Combined with evidence that older homeless adults experience age-related health conditions^2^ at earlier ages than the general population, have higher rates of hospital-based care,^6^ and enter nursing homes at higher rates than their housed peers,^5^ our findings underscore the urgency with which policy and programmatic responses to address the needs of this population should be deployed.

## Notes

Supplement.
Data Sharing Supplement
