# Supplementary material for: Persistence of a Birth Cohort Effect in the US Among the Adult Homeless Population
Source: JAMA Netw Open. 2024 Dec 26;7(12):e2452163. doi: 10.1001/jamanetworkopen.2024.52163 (PMC11672155; doi:10.1001/jamanetworkopen.2024.52163)
Supplement: Supplement. — Data Sharing Supplement [file jamanetwopen-e2452163-s001.pdf]

Data Sharing Statement

Byrne. Persistence of A Birth Cohort Effect in the US Among the Adult Homeless Population. *JAMA Netw Open*. Published December 26, 2024. doi:10.1001/jamanetworkopen.2024.52163

Data
Data available: No
